# Aberrant Activity of Histone–Lysine N-Methyltransferase 2 (KMT2) Complexes in Oncogenesis

**DOI:** 10.3390/ijms21249340

**Published:** 2020-12-08

**Authors:** Elzbieta Poreba, Krzysztof Lesniewicz, Julia Durzynska

**Affiliations:** 1 Institute of Experimental Biology, Faculty of Biology, Adam Mickiewicz University, ul. Uniwersytetu Poznańskiego 6, 61-614 Poznań, Poland; 2 Department of Molecular and Cellular Biology, Institute of Molecular Biology and Biotechnology, Faculty of Biology, Adam Mickiewicz University, ul. Uniwersytetu Poznańskiego 6, 61-614 Poznań, Poland; lesniew@amu.edu.pl

**Keywords:** histone–lysine N-methyltransferase 2, COMPASS, COMPASS-like, H3K4 methylation, oncogenesis, cancer, epigenetics, chromatin

## Abstract

KMT2 (histone-lysine N-methyltransferase subclass 2) complexes methylate lysine 4 on the histone H3 tail at gene promoters and gene enhancers and, thus, control the process of gene transcription. These complexes not only play an essential role in normal development but have also been described as involved in the aberrant growth of tissues. KMT2 mutations resulting from the rearrangements of the KMT2A (MLL1) gene at 11q23 are associated with pediatric mixed-lineage leukemias, and recent studies demonstrate that KMT2 genes are frequently mutated in many types of human cancers. Moreover, other components of the KMT2 complexes have been reported to contribute to oncogenesis. This review summarizes the recent advances in our knowledge of the role of KMT2 complexes in cell transformation. In addition, it discusses the therapeutic targeting of different components of the KMT2 complexes.

## 1. Introduction

In the past two decades, efforts have been made to create a comprehensive and universal description of common traits of cancer cells. This gave rise to a new term—cancer hallmark—that reflects each of the intrinsic characteristics of a cancer cell not found in normal cells. Although a vigorous debate is still taking place, it can be agreed upon that cancer cells are independent of growth and antigrowth stimuli. These cells can evade apoptosis and replicate in a limitless manner and, hence, become immortal. Cancer cells also induce angiogenesis for a better supply of nutrients and are capable of spreading to different sites of the body (metastasis). Furthermore, they can reprogram energy metabolism, escape the immune response system, and promote inflammation. The underlying conditions are mutations and genome instability, which allow for the establishment of all cancer hallmarks [1,2,3,4]. However, the genetic landscape of cells undergoing transformation is only one part of the story, as the acquired knowledge of epigenetic modifications has helped us understand their crucial importance and contribution to virtually all classic hallmarks of cancer [5]. Recent studies based on the high-throughput sequencing of genomes, epigenomes, and transcriptomes have provided us with enormous data pointing to the relationship between cancer development and mutations in genes encoding epigenetic modifiers. Abnormal histone methylation resulting from gene mutation, translocation, or the deregulated expression of histone methyltransferases has been frequently observed in cancers. Depending on the histone and the modified residue, the process of methylation plays various biological roles. The methylation of lysine 4 on histone H3 is associated with transcriptional activation and is catalyzed by histone methyltransferases classified as KMT2s (lysine-specific methyltransferases subclass 2) [6]. H3K4 trimethylation (H3K4me3) is characteristic of accessible gene promoters, whereas H3K4 monomethylation (H3K4me1) occurs in enhancer elements [7,8,9].

This review aims to present the most recent advances in our understanding of the structure and role of KMT2 complexes in cellular transformation. The oncogenic outcomes of mutations in KMT2s, as well as in the core subunits of the KMT2 complexes, are discussed. In addition, a number of molecular strategies targeting different components of the KMT2 complexes with anticancer therapeutic potential are described in detail.

## 2. Structure of the KMT2 Complexes

**Figure 1:**
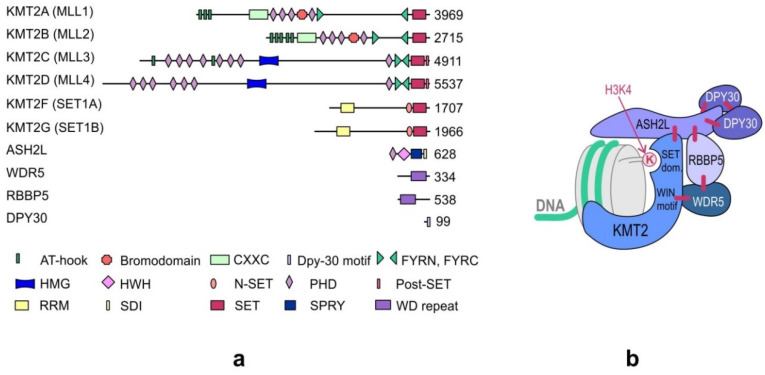
(**a**) Domain structure of the KMT2 family and core subunits of the KMT2 complexes. The numbers indicate the number of amino acids. KMT, histone–lysine N-methyltransferase; ASH2L, absent, small, or homeotic 2-like; DPY30, Dumpy-30; RBBP5, retinoblastoma-binding protein 5; WDR5, WD repeat-containing protein 5; AT-hook, adenosine-thymidine-hook; CXXC, Zinc finger-CXXC domain; FYRN/FYRC, phenylalanine and tyrosine-rich region (N- and C-terminal); HMG, high mobility group; HWH, helix-wing-helix domain; N-SET, N-terminal of SET; PHD, plant homeodomain; Post-SET, C-terminal of SET; RRM, RNA recognition motif; SDI, Sdc1-Dpy-30 interaction; SET, Su(var)3-9, Enhancer-of-zeste and Trithorax; SPRY, SPla and the ryanodine receptor domain; and WD repeat, tryptophan-aspartic acid repeat. (**b**) The structure of the KMT2 complex. The enzyme and the core subunits of the complex are shown in the diagram. The interactions between individual subunits are marked with blue lines. Subunits specific to individual KMT2 complexes, not shown in the figure, interact with the amino terminus of the KMT2s. WIN motif, WDR5 interaction motif.

KMT2s are the main H3K4 methyltransferases that regulate gene transcription. The KMT2 family is highly conserved in eukaryotes. While three subgroups of KMT2s, each with a single representative, are present in *Drosophila melanogaster*—trithorax (Trx), trithorax-related (Trr), and Set1—two paralogs of each subgroup appeared in humans during evolution. Human cells contain two Trx-related KMT2s (KMT2A and KMT2B), two Trr-related KMT2s (KMT2C and KMT2D), and two Set1-related KMT2s (KMT2F and KMT2G) (Figure 1). Although KMT2E was initially classified under the KMT2 family, it was identified to be more homologous to yeast SET3 (SET domain-containing protein 3) and SET4 (SET domain-containing protein 4) and *Drosophila* CG9007 (encoding the protein “UpSET”) [10,11,12]. Moreover, unlike the other KMT2s present in humans, KMT2E does not exhibit intrinsic methyltransferase activity toward histone substrates [12].

The KMT2s function in large multi-subunit complexes, which, in vertebrates, are often referred to as COMPASS or COMPASS-like complexes (COMplex of Proteins ASsociated with Set1). While there is one COMPASS complex in yeast, there are three in *Drosophila* and six closely related complexes in vertebrates, which contain one KMT2 methyltransferase unique to each complex, four core subunits commonly found in all KMT2 complexes, and additional complex-specific proteins (Table 1).

The four core subunits—WDR5 (WD repeat domain 5), RBBP5 (retinoblastoma-binding protein 5), ASH2L (absent, small or homeotic 2-like), and DPY30 (Dumpy-30)—form a subcomplex that stably interacts with the KMT2 enzymes and stimulates KMT2 catalytic activity up to several hundred-fold [13,14]. A crystal structure analysis of the KMT2A complex has shown that the interaction of KMT2A with the core subunits (WDR5, RBBP5, and ASH2L) forces a conformational change in the SET domain of KMT2A, which is necessary to achieve a catalytically efficient form [14,15,16,17]. Biochemical and structural studies have demonstrated that the KMT2A complex is stabilized by direct interactions between KMT2A and WDR5 that bridges KMT2A to RBBP5. Furthermore, RBBP5 interacts with ASH2L, which binds the DPY30 protein. Although WDR5 is essential for regulating the activity of KMT2A, it is not responsible for the regulation of other KMT2s, and these KMT2s have been shown to be stimulated by the stable ASH2L-RBBP5 heterodimer that directly interacts with them [13]. The structure of the KMT2 complex is presented in Figure 1b.

In addition to their role in stabilizing and regulating KMT2 complexes, the core subunits of the WRAD subcomplex participate in the recruitment of these complexes to chromatin. The recruitment of KMT2 complexes to the genomic loci is also regulated by the unique complex-specific subunits that determine the functional diversity of these complexes. The three KMT2 groups of complexes differ in their sets of interacting proteins (Table 1). The additional subunits associated with the KMT2A/KMT2B complexes are Menin and HCF1/2 (host cell factors 1/2) [18,19]. Another protein that interacts with the KMT2A/KMT2B complexes is LEDGF (lens epithelium-derived growth factor, also known as PSIP1/p75). However, LEDGF is a substoichiometric component in these complexes and exhibits only a weak interaction with them through Menin [19,20,21]. PTIP (PAX transactivation domain-interacting protein), PA1 (PTIP-associated 1), NCOA6 (nuclear receptor coactivator 6), and UTX (ubiquitously transcribed tetratricopeptide repeat, X chromosome) interact specifically with KMT2C and KMT2D [22,23,24], while CFP1 (CXXC finger protein 1), WDR82 (WD repeat domain 82), and HCF1 (host cell factor 1) interact only with KMT2G and KMT2F [19,25].

In addition to the stable interaction with the complex-specific subunits, the KMT2 complexes dynamically interact with many transcription factors, including NFE2 (nuclear factor, erythroid 2) [26], USF1 (upstream transcription factor 1) [27], MEF2D (myocyte enhancer factor 2D) [28], NF-Y (nuclear transcription factor Y) [29], NF-E2 (nuclear transcription factor erythroid 2) [26,30], AP2δ (activating protein 2δ) [31], MYC [32], OCT4 (octamer-binding transcription factor 4, also known as POU5F1) [33], NANOG [34], p53 [35,36,37], E2Fs [35,38,39], and PAX7 (Paired Box 7) [40]. These interactions of the KMT2 complexes are important for their context-dependent roles.

## 3. Domain Structure of KMT2s

All KMT2s contain an evolutionarily conserved catalytic SET domain. The acronym “SET” is derived from the first three proteins identified as harboring this domain in *Drosophila*—Su(var)3-9, Enhancer-of-zeste, and Trithorax [41]. SET domains catalyze the transfer of methyl groups from S-adenosyl-L-methionine onto the Nε group of a lysine in substrate proteins. Unlike other proteins in the KMT2 family, KMT2A and KMT2B have the consensus sites cleaved by taspase 1, a threonine aspartase [38,42]. This cleavage generates two fragments of KMT2A/KMT2B proteins: a large amino-terminal fragment and a smaller carboxy-terminal fragment. These two fragments associate through the FY-rich N-terminal (FYRN) and FY-rich C-terminal (FYRC) domains to form a functional heterodimeric complex.

KMT2s contain specific domains for interaction with DNA, histones, and other proteins. KMT2A–KMT2D contain clusters of plant homeodomain (PHD) fingers, which are one of the epigenetic readers of H3K4me3. Although many PHD fingers are present in KMT2s, only PHD3 of KMT2A has been shown to bind H3K4me3 [43]. The other PHD fingers do not recognize methylated H3K4 and are instead involved in recognizing other histone modifications [44]; for example, PHD4–6 of KMT2D recognize nonmethylated or asymmetrically methylated arginine 3 of histone H4. Binding to H4R3me2a is important for the nucleosomal methyltransferase activity and localization of KMT2D. The PHD fingers of KMT2s also interact with other proteins. For example, PHD3 of KMT2A interacts with cyclophilin 33 (CYP33 or PPIE) [45]. CYP33 was shown to compete with PHD3 for binding with H3K4me3, thereby leading to the repression of KMT2A target genes [46]. PHD2 fingers of KMT2A and KMT2B have E3 ubiquitin ligase activity and were shown to ubiquitinate H3 and H4 histones in vitro [47]. The loss of PHD fingers in KMT2A due to genomic translocation in MLL-r leukemia is responsible for the transforming potential of the resulting fusion proteins, which indicates the role of PHD fingers in the correct recruitment of KMT2A into the genomic loci [48,49]. Furthermore, the CXXC zinc finger domain, the AT-hook region, and the high-mobility group I (HMG-I)-binding motif present in some KMT2s are involved in DNA binding. KMT2A-KMT2D contain LXXLL motifs (also known as NR boxes), characteristic for transcription factors and cofactors interacting with nuclear receptors. The number of NR boxes varies in different MLLs [50]. A characteristic future of the protein sequence of KMT2F and KMT2G is the presence of the C-terminal N-SET domain, which interacts with the ubiquitylated histone H2B and an RNA recognition motif (RRM). The domain structure of KMT2s is presented in Figure 1a.

## 4. Substrate Specificity of KMT2 Complexes and Their Recruitment to Chromatin

**Figure 2:**
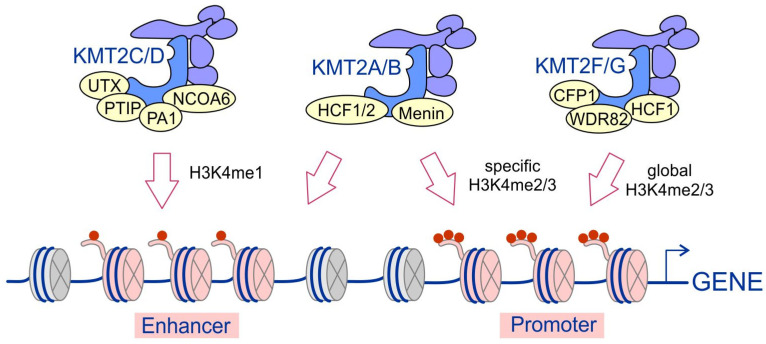
Specialization of COMPASS and COMPASS-like complexes in mammals. The KMT2F/KMT2G complexes, referred to as COMPASS complexes, are most similar to the yeast Set1 complex. These complexes are mainly responsible for *bulk* H3K4me3 genome-wide. The KMT2A/KMT2B and KMT2C/KMT2D complexes are referred to as COMPASS-like and are most similar to the *Drosophila* Trithorax complex and *Drosophila* Trithorax-related protein, respectively. KMT2A/KMT2B complexes are required for the H3K4 tri- and dimethylation in less than 5% of promoters and mainly regulate developmental genes. KMT2C/KMT2D complexes occupy enhancers and are responsible for H3K4 monomethylation.

The KMT2 complexes differ in their specificity and genome-wide localization. Differences in their unique subunit composition and their interactions with transcriptional regulators determine the location of these complexes throughout the genome (Figure 2). The KMT2F/KMT2G complexes are highly enriched at gene promoters and responsible for the vast majority of H3K4 di- and trimethylation processes [27,51]. They are localized to distinct regions of chromatin and generally act in a nonredundant manner [25]. It was recently shown that KMT2F and KMT2G are responsible for broad H3K4me3 peaks and that KMT2G is functionally redundant to KMT2F at highly expressed genes in embryonic stem (ES) cells [52]. The KMT2A/KMT2B complexes are also important for H3K4 di- and trimethylation, but ChIP experiments showed that less than 5% of global H3K4 trimethylation is KMT2A-dependent, and one of the well-characterized targets of KMT2A was identified to be *HOX* genes [53]. Moreover, KMT2A/KMT2B complexes have been shown to bind to de novo enhancers [54]. In contrast, the KMT2C and KMT2D complexes are responsible for H3K4 monomethylation (H3K4me1) and occupy enhancers enriched with this type of modification [54,55,56].

**Figure 3:**
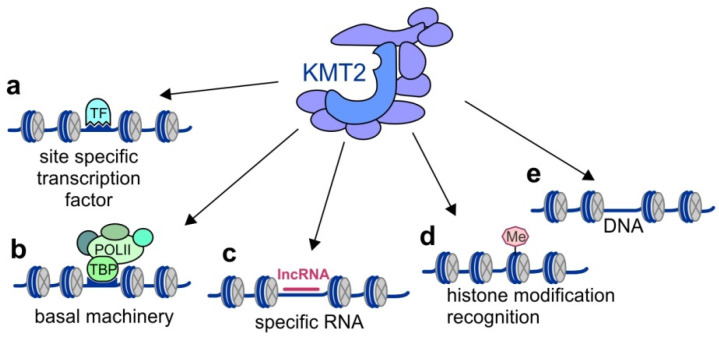
Mechanisms of H3K4 methyltransferase recruitment to chromatin. KMT2 complexes are recruited and stabilized on chromatin by a combination of mechanisms: (**a**) interactions with sequence-specific transcription factors, (**b**) association with the basal transcriptional machinery, (**c**) interaction with long noncoding RNAs (lncRNAs), (**d**) recognition of histone modification, and (**e**) direct interaction with DNA. For references, see the text.

The recruitment of KMT2 complexes to specific genomic loci is mediated by multiple mechanisms, including the interaction of these complexes with sequence-specific transcription factors, basal transcription factors, cofactors, and chromatin specified by histone modifications and the mechanisms of long noncoding RNAs (lncRNAs) (Figure 3). Both KMT2 core subunits and additional complex-specific subunits, as well as KMT2s, have been shown to interact with different transcription factors. For example, Menin, a unique subunit in KMT2A/KMT2B complexes, mediates their recruitment to specific genomic loci by interacting with estrogen receptor-α (ERα) [57]. KMT2C and KMT2D interact through PTIP with the PAX family of transcription factors, including Pax2 (Paired Box 2) and Pax5 (Paired Box 5) [22,24], and through NCOA6 (ASC-2) with nuclear receptors such as the farnesoid X receptor (FXR), which regulates the genes involved in bile acid homeostasis [58], as well as with the MAFA (MAF BZIP Transcription Factor A) and MAFB (MAF BZIP Transcription Factor B) transcription factors to regulate the functions of islet beta cells [59]. In addition, KMT2C/KMT2D complexes may be recruited by the tumor suppressor p53 by interaction with 53BP1 that binds to NCOA6 [36]. The subunits of the WRAD subcomplex also interact with many transcription factors. For instance, WDR5 binds to the pluripotency transcription factor OCT4 and recruits KMT2 complexes to mediate self-renewal and pluripotency of the ES cells [33]. OCT4 has also been shown to interact directly with KMT2F independently of WDR5 [51] and with ASH2L through SOX2. This recruitment mechanism of the KMT2 complexes was reported to be important for efficient cellular reprogramming of differentiated cells into induced pluripotent stem (iPS) cells [60]. Furthermore, the recruitment of these complexes onto the key developmental genes in ES cells is mediated by the core pluripotency factor NANOG that interacts with DPY30. NANOG cooperates with the activin-SMAD2/3 signaling pathway and was shown to coordinate the fate decisions of human ES cells by controlling H3K4me3 [34]. Another transcription factor that interacts with the KMT2 complexes is MYC. This interaction is mediated by ASH2L and has been found to control H3K27 modifications and regulate bivalent chromatin [32]. MYC interacts not only with ASH2L but, also, with WDR5. However, the interaction with WDR5 renders MYC recruitment to chromatin independent of other WRAD subunits [32,61]. The recruitment of the KMT2 complexes through ASH2L for controlling H3K4 methylation has also been shown for other transcription factors, such as USF1 (upstream transcription factor 1) [27], NF-Y (nuclear transcription factor Y) [29], NF-E2 (nuclear transcription factor erythroid 2) [26,30], MEF2D transcription factor [62], and Ap2delta (TFAP2D) [31,63].

Furthermore, KMT2 complexes can interact with RNA polymerase (RNAP) II and basal transcription factors. For example, WDR82 was found to directly interact with RNAP II phosphorylated at Ser 5 and mediate the recruitment of the KMT2F complex to transcription start sites [64]. KMT2A was shown to interact with RNAP II through an RNAP II-associated factor, hPaf1/PD2 [65].

Recent data indicate that the recruitment of KMT2 complexes to chromatin can also be mediated through their interactions with lncRNAs, which may act as a scaffold guiding the KMT2 complexes to specific genomic loci [66]. KMT2 complexes bind to lncRNAs via the WDR5 subunit, which, in turn, has been shown to interact with many lncRNAs through an RNA-binding pocket. The interaction of WDR5 with lncRNA HOTTIP (HOXA transcript at the distal tip), which regulates the expression of the *HOXA* gene cluster, was observed to be involved in the recruitment of WDR5/KMT2A complexes to the *HOXA* locus and, thus, promote H3K4 trimethylation and *HOXA* gene transcription. The expression of the *HOXB* cluster is also regulated by the interaction of KMT2s with a lncRNA. The HoxBlinc lncRNA regulating the *HOXB* cluster interacts with the SET domain of the KMT2A or KMT2F enzyme. This mechanism activates the anterior *hoxb* genes and, thus, promotes the differentiation of cardiogenic/hemogenic mesoderm [67].

KMT2 complexes can also directly interact with DNA, which allows them to function independently of transcription factors at some genomic loci. KMT2 methyltransferases contain DNA-binding domains, including the CXXC zinc finger domain, the AT-hook region, and the HMG-I-binding motif. The CXXC zinc finger domains present in KMT2A and KMT2B bind to nonmethylated CpG-containing DNA sequences [68,69,70,71]. The CXXC domain is absent from other KMT2 methyltransferases. However, it can be found in the CFP1 protein, a subunit specific to the KMT2F/KMT2G complexes, and is likely used by them for interacting directly with CpG-rich DNA [72,73]. The AT-hooks and HMG-I are small motifs that bind to the minor groove of AT-rich DNA sequences in a distorted or cruciform conformation [74]. The DNA-binding motifs present in KMT2 methyltransferases stabilize them on chromatin. Their functional importance has been demonstrated in structure–function studies, which showed that the DNA-binding domains, AT-hooks, and CXXC domain present in KMT2A are responsible for the transforming ability of the KMT2A fusion protein [75].

**Figure 4:**
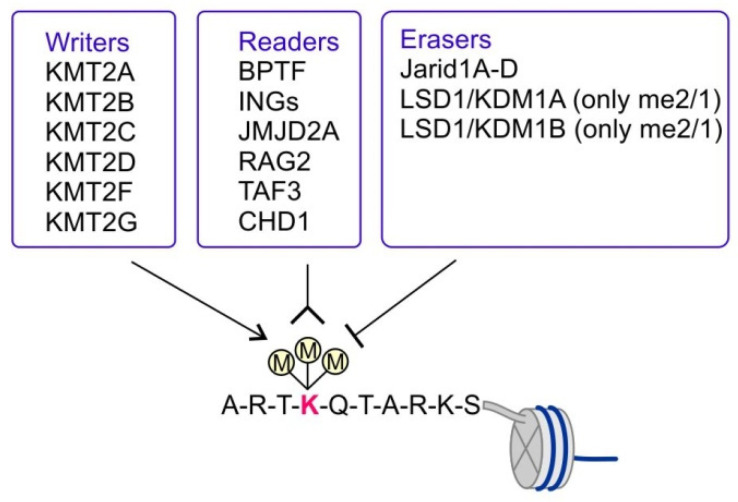
Known writers, readers, and erasers of H3K4 methylation.

H3K4 methylation is recognized by reader proteins that transfer the information related to epigenetic modifications to the basal transcription machinery, thus promoting transcription. Several methylated H3K4 interactors have been identified so far (Figure 4). These interactors bind to methylated H3K4 via different domains, including PHD, tandem Tudor domain (TTD), double chromodomains (DCD), zinc finger CW (zf-CW), and the recently discovered CryptoTudor domain [8,76,77,78]. A PHD domain capable of binding to H3K4me3 was identified, for example, in TAF3 (TBP-associated factor 3), a TFIID component that plays an important role in the recruitment of the RNAP II complex to core promoters [79,80]. The chromatin remodeling complex NURF (nucleosome remodeling factor) is another important reader of H3K4me3 [81,82]. BPTF (Bromodomain PHD Finger Transcription Factor), a subunit of the NURF complex, has been shown to bind to H3K4me3, thereby coupling H3K4me3 to chromatin remodeling, as well as to maintain the patterns of *HOX* gene expression during development [82]. Other noteworthy proteins that are capable of reading the H3K4me3 modification include the ING (inhibitor of growth) family of proteins [83,84,85] and PHF13 [86] proteins. The CryptoTudor domain identified in the BRWD2/PHIP protein has been shown to interact both with H3K4me1 and KH3K4me3 [87]. BRWD2/PHIP was found at both enhancers marked by H3K4me1 and promoters enriched in H3K4me3 and is likely to control H3K27ac in bivalent chromatin [87].

Although the main substrate of the KMT2 complexes is lysine 4 of histone 3, nonhistone substrates of these enzymes have also been identified. KMT2F has been shown to methylate the HSP70 and YAP proteins, thus promoting tumorigenesis [88,89]. A recent study demonstrated that p53 may be a potential substrate of KMT2 family methyltransferases [90]. KMT2A-D and KMT2G have been shown to be capable of methylating p53 at K305 in in vitro tests; however, the physiological role of this modification has not yet been elucidated.

## 5. Mutations in the KMT2 Family in Cancer

The relationship between KMT2 complexes and oncogenesis was first demonstrated by the example of *KMT2A*, which is translocated to other fusion gene partners in approximately 10% of human leukemias [91]. Recent studies have shown that KMT2 mutations not only influence the development of neoplastic processes in leukemias but are also among the most common changes observed in human cancers and are associated with different types of solid tumors.

### 5.1. KMT2A (MLL1) and KMT2B (MLL2)

**Figure 5:**
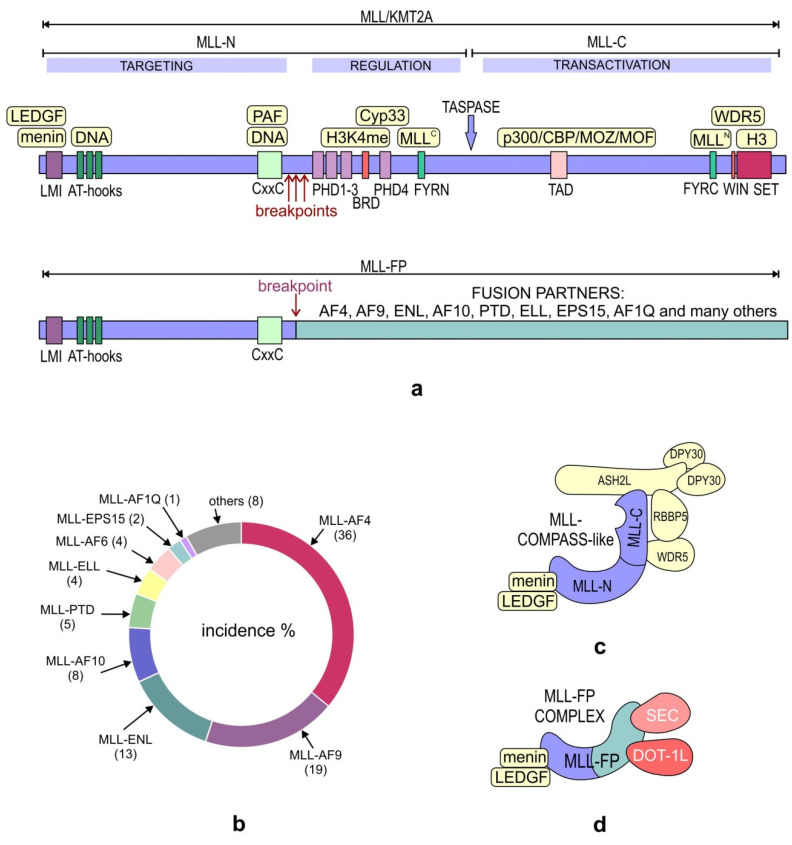
Wild-type KMT2A (MLL) and diverse KMT2A-fusion proteins (MLL-FPs). (**a**) Schematic representation of the wild-type (wt) KMT2A structure. Each domain is labeled in black capital letters, while interacting proteins are marked in rounded yellow-shaded rectangles. A cleavage site recognized by taspase 1 is annotated with a blue arrow. Three major functional parts of the protein responsible for targeting, regulation, and transactivation are also indicated. The protein product of the *MLL/KMT2A* gene is composed of 3969 amino acids and contains many domains that are involved in the regulation of gene transcription [122]. These include an AT-hook motif-binding DNA [123], a CXXC domain rich in cysteines [124,125], homeodomain (PHD) finger motifs [126], a bromodomain (BRD) [127], and a potent transcriptional activation domain (TAD) located between the amino acids 2829 and 2883 [128,129]. The KMT2A protein also contains a WIN motif responsible for the interaction with WDR5 and maintaining the H3K4me2 activity of the MLL core complex in vitro [130,131,132]. At the carboxy-terminal part of the protein, there is a SET domain conferring the histone methyltransferase activity of MLL [133,134]. A natural maturation course of MLL includes its proteolysis into two fragments, MLL-N and MLL-C, which then form a complex in vivo [135,136]. Two motifs—namely, FYRC and FYRN—are indispensable for heterodimerization between the terminal fragments of cleaved MLL, which can interact with many other proteins to form multiunit complexes required for transcriptional coactivator activity [134,137]. In MLL-FPs, many N-terminal domains, such as PHD, FYRN, FYRC, and SET, are lost. A detailed description is provided in the main text. (**b**) The most frequent rearrangements of KMT2A (MLL) in MLL. The data were obtained from Meyer et al. [99]. The three most frequent translocations of MLL are with AF4, AF9, and ENL. (**c**) Composition of the wt MLL COMPASS-like complex containing MLL-N and MLL-C. Core subunits (DPY30, ASH2L, WDR5, and RBBP5); Menin; and LEDGF are shown. (**d**) Composition of an MLL-FP complex. It contains MLL-FP, DOT1L, SEC, Menin, and LEDGF—the last two proteins are present in both wt and rearranged complexes.

Originally, the first somatic mutations linked to cancer development were described for the *KMT2A* gene located on chromosome 11q23 [92], which is rearranged with a considerable number of other translocation partner genes (TPGs) [93,94,95]. This translocation is possible because of frequent chromosomal double-stranded DNA (dsDNA) breaks that are not repaired in developing hematopoietic cells [96]. All KMT2A fusion proteins are coded by the first 8 to 13 exons of the *KMT2A* gene making up the C-terminal part of the hybrid protein and a variable number of fusion partner (FP) exons coding for the N-terminal part [97]. Normally, as a part of the COMPASS-like complex, KMT2A methylates H3K4 through its SET domain and, thus, regulates the transcription of target genes [98]. After the translocation of *KMT2A* to its FP, this domain is lost in all MLL-FPs. As of 2018, a total of 135 different *KMT2A* rearrangements have been identified, the majority of which are in-frame translocations resulting in functional gain-of-function (GOF) oncoprotein products displaying altered activities [99]. However, there are several most common genes fused with the *KMT2A* gene, accounting for an overwhelming majority of mixed-lineage leukemias (MLLs; 80–90%), including MLL-AF4, MLL-AF9, MLL-ENL, MLL-PTD, MLL-AF10, and MLL-ELL [99,100,101,102,103] (Figure 5a,b). On the other hand, MLL-rearranged (MLL-r) leukemias account for approximately 10% of all human leukemias [104]. Fusions with TPGs such as AF4, AF9, AF10, and ENL lead to the formation of MLL-r complexes, in which MLL-FPs directly interact with the H3K79 methyltransferase DOT1L (disruptor of telomeric silencing 1-like) and super elongation complex (SEC). These interactions lead to aberrant H3K79 methylation, thereby reshaping the gene expression pattern toward leukemic transformation [105,106,107,108,109]. Quite similarly, the MLL-ENL chimeric protein, along with its interacting proteins, including DOT1L, is capable of methylating H3K79 [110]. Due to *KMT2A* gene fusion, other histone amino acids can also be modified; for example, MLL-EEN, together with CBP (CREB-binding protein) and PRMT1 (protein arginine N-methyltransferase 1), methylate H3R4 [111]. Another interesting shift in the epigenetic activity is the direct translocation of *KMT2A* to *CBP* or *P300* responsible for the histone acetyltransferase activity of the fusion proteins [112,113,114]. In general, either a new protumorigenic epigenetic activity can be linked to altered interacting partners of MLL-FPs such as DOT1L, which are not present in wild-type (wt) KMT2A H3K4 methyltransferase COMPASS-like complexes, or a part of KMT2A is directly fused with a protein bearing a different modifying activity, such as CBP. A reverse fusion was also demonstrated as possible—namely, AF4-MLL—which resulted in a protein product having the capacity to induce acute lymphoblastic leukemia (ALL) by promoting transcription elongation in mice or murine hematopoietic progenitor/stem cells through interactions with DOT1L, P-TEFb (Pol II-positive transcription elongation factor), and other core subunits of the MLL-FP complexes. This positive regulation of RNAP II by P-TEFb allows for productive transcription and the synthesis of proteins involved in cell transformation [115,116,117]. It has been shown that wt KMT2A is essential for leukemogenesis and helps maintain the cells that were transformed due to the action of MLL-AF9. An important role in this process is played by Menin, which associates with both wt KMT2A and MLL-AF9 to recruit them to *HOX* genes for activating their transcription. Apart from catalyzing the methylation of H3K4, wt KMT2A reinforces MLL-AF9-dependent H3K79 methylation at the target genes such as *HOX* genes, as it was demonstrated that H3K79me was reduced at this locus in the wt KMT2A knockdown cells. The wt allele also upregulates pro-proliferating cell cycle genes such as *CCNA2*. It can be concluded that both alleles, the wt and rearranged ones, are crucial in the oncogenic transformation of blood cells [118]. It is of note that the capacity of MLL-FPs to transform hematopoietic stem cells is dose-dependent. These chimeric proteins facilitate self-renewal, enhance the expression of antiapoptotic genes, and induce the synthesis of drug efflux pumps, all of which are inherent properties of stem cells [97,119]. Such leukemia stem cell-specific transcription programs are achieved by the MLL-FP complexes that directly target genes such as *HOXA9*, *HOXA10*, and *MEIS1* [120,121]. The composition of the wt MLL and MLL-FP complexes is presented in Figure 5c,d.

The majority of MLL cases are very young patients under one year of age suffering from ALLs, while a few are young- to middle-aged adults developing acute myeloid leukemias (AMLs). There is also a third group of patients who are much rarer and treated with chemotherapeutic agents, the side effects of which can induce the so-called therapy-related leukemia [138]. One of the drugs targeting the DNA topoisomerase II induces 11q23 translocations and leads to the treatment-related development of AML [139,140]. It should be stressed here that most *KMT2A* translocations have been linked to a poor prognosis in patients with MLLs [141]. The term “mixed-lineage leukemia” reflects an observation that, during chemotherapy, ALL patients may relapse as AML patients and that myeloid/lymphoid phenotypes can be reversed in some cases [142,143]. This is supported by the fact that around 5% of ALLs and 5–10% of AMLs in adults are due to MLL rearrangements [97,144].

*KMT2A* gene rearrangements in leukemias have been studied for the longest time [97]. Nevertheless, new fusions are still being found, including the ones very recently described in solid tumors such as sarcomas [145]. Other types of *KMT2A* mutations have also been reported, which include tandem duplications and gene amplifications, as well as mutations in the coding region, such as missense, nonsense, and frameshift ones [146]. Although these mutations are not really frequent in leukemias, they appear at a higher incidence in numerous solid tumors [147,148,149,150]. Most of the *KMT2A* mutations induce the production of truncated proteins with a lost SET domain activity, while others can affect the N-terminal part of the protein, which is devoid of PHD, CXXC, or the WDR5-interacting domain [146]. In the vast majority of cancers, all the reported *KMT2A* mutations are heterozygous, which means that at least one wt *KMT2A* allele is not altered. The loss of one *KMT2A* allele is insufficient to trigger leukemogenesis or induce cell transformation in other tissues, as demonstrated by genetic studies in mice. The same lack of predisposition to cancer development was observed in patients with haploinsufficient germline *KMT2A* mutations (one allele is mutated, one is not, and the concentration of the gene product is decreased). This indicates that, on the one hand, leukemic transformation can be driven by the GOF *KMT2A* translocations producing MLL-FPs, while, on the other hand, the development of cancers carrying KMT2A loss-of-function alterations most probably need additional transforming events independent of KMT2A. Whether KMT2A mutations have the potency to drive cell transformation or act only as a permissive background for oncogenesis is a question that remains to be answered [146].

Accumulating data demonstrate that KMT2A can play various roles in the development and maintenance of cancers. As mentioned earlier, wt *KMT2A* is important to sustain the leukemic state of cells transformed by the actions of some MLL-FPs. The presence of wt *KMT2A* is also crucial in other types of cancers in which KMT2A is overexpressed. For example, the knockdown of KMT2A in cervical cancer cells (HeLa) using antisense RNA resulted in increased apoptosis. This effect, to a lesser extent, was also observed in other cancer cells but not in nonmalignant ones. This suggests that cancer cells are more sensitive than normal cells to the loss of KMT2A activity, which is required to maintain their oncogenic phenotype. Moreover, it was demonstrated that KMT2A, together with hypoxia-inducible factor 1α (HIF1α), were overexpressed in tumor regions with reduced oxygen levels. The KMT2A knockdown influenced angiogenesis by reducing the expression of HIF1α and vascular endothelial growth factor (VEGF) in cervical cancer xenografts. Additionally, it led to the suppression of xenografted tumor growth in vivo. These data suggest that KMT2A acts as an important factor in hypoxia signaling, vasculogenesis, and tumor growth [151]. Another study reported an increased activity of KMT2A and the global distribution of an H3K4me3 mark at promoter sequences in the salivary gland and head and neck squamous cell carcinomas, which were dependent on the high activity of Wnt-β-catenin signaling [152]. A very interesting recent study shed more light on how crucial the role of KMT2A is in these solid tumors. GOF Wnt/β-catenin increased KMT2A activity, leading to high H3K4me3 at the promoters of *Axin2*, *Id2*, *Tcf7l2*, *Tiam1*, and *Ephb3*, which are well-established Wnt target genes. By the genetic ablation of *KMT2A* (CRISPR/Cas9), as well as mutations of the SET domain or the pharmacological inhibition of important protein–protein interactions of the KMT2A complex, it was shown that the H3K4me3 mark was reduced in tumor cells, and the sizes of tumor organoids and tumor spheres were greatly diminished [153]. An increased *KMT2A* expression was also found in solid tumors with GOF mutations of *TP53* [154]. In these cancers, p53 mutants bound to and upregulated the genes encoding KMT2A, KMT2D, and acetyltransferase MOZ (KAT6A), resulting in the global enhancement of H3K4 methylation and histone acetylation, as well as upregulation of the KMT2A target genes, including the *HOXA* gene cluster. The *KMT2A* knockdown or pharmacological inhibition of KMT2A were sufficient to inhibit tumor progression, which indicates that KMT2A is essential for the cancer phenotype of cells containing GOF p53 mutants and for driving cancer growth.

KMT2B is generally considered to positively regulate cell growth [155], and homozygous *KMT2B* inactivation in ES cells leads to defects in cell proliferation and apoptotic outcome [155,156]. A region harboring the *KMT2B* gene was shown to be amplified in certain pancreatic cancer cells and was frequently translocated in glioblastomas [157]. Similar to *KMT2A*, *KMT2B* haploinsufficiency in mice, whether germline or conditional, did not show any potency to initiate oncogenesis [155,158,159]. Moreover, *KMT2B* carries similar mutations in cancers, which are predominantly missense, nonsense, and frameshift affecting the SET and PHD domains [146]. *KMT2B* displays higher mutation rates in uterine corpus endometrial carcinoma (UCEC), as demonstrated by the next-generation sequencing approach [160], and in gastric cancer [161] and esophageal sarcomatoid carcinoma [162]. A recent study demonstrated in a young man with NF1-GBM (neurofibromatosis 1-glioblastoma) that somatic mutations in *KMT2B* leading to protein truncation were early driver events of malignant gliomagenesis, whereas other pan-cancer genes were mutated later, as revealed by whole-genome sequencing of the patient’s brain samples analyzed at different times of the disease development [163].

As far as the role of the wt KMT2B in MLL-r leukemia is concerned, it was found to be crucial in the maintenance of MLL-AF9-induced AML cells. Deletion of the wt *KMT2B* was shown to reduce the overall leukemic cell survival, while the deletion of *KMT2A* did not affect the MLL-r cells [164]. This observation is in contrast to that described earlier and suggests that the activities of KMT2B and KMT2A could be redundant and substitute each other in some cases of MLL-r leukemia [118].

### 5.2. KMT2C (MLL3) and KMT2D (MLL4)

Unlike KMT2A and KMT2B, KMT2C and KMT2D are known to impede cell proliferation and, oftentimes, are considered as tumor suppressors [36,160,165,166,167,168]. Studies based on chromosome aberration mapping established that the genomic regions containing the *KMT2C* and *KMT2D* genes were associated with cancer. Deletion of the region containing the *KMT2C* gene was common in AMLs [169]. Although KMTs were initially studied for their causative role in hematological malignancies resulting from KMT2A rearrangements, we know today that *KMT2C* and *KMT2D* are the most frequently mutated among the human cancer genes [160,170,171,172].

KMT2C and KMT2D share the same types of mutations, mainly nonsense and frameshift alterations in the SET and PHD domains, resulting in a truncated version of the synthesized protein products. Missense alterations in the SET domains of KMT2C and KMT2D have also been reported but very rarely [146]. In addition, a mutation hotspot within the PHD cluster of the *KMT2C* gene was described, the mutations of which disrupted the interaction of KMT2C with the BAP1 (BRCA1 associated protein-1) tumor suppressor, resulting in poor patient survival in many types of cancer (liver, lung, bladder, and breast). As for KMT2D, the mutations were more evenly distributed across the entire gene [173,174]. Both *KMT2D* and *KMT2C* genes were reported to be mutated with an increased frequency in bladder urothelial cell carcinoma (BUCC) [160]. *KMT2C* is frequently inactivated by deletion in myeloid leukemia [169] and, also, carries somatic mutations in the interdomain regions in glioblastoma multiforme and pancreatic ductal adenocarcinoma (PDAC) [175]. Moreover, the gene is frequently mutated in diffuse-type gastric adenocarcinoma (DGA). The loss of expression of *KMT2C* in DGA promotes epithelial–mesenchymal transition (EMT) and is linked with worse overall survival [176]. A study showed that *KMT2D* was mutated in 8.6% of prostate cancer cases, while the genes of other MLLs had altered copy numbers or had mutations [177]. In another study, *KMT2C* was several times more frequently mutated (~7% of cases) compared to *KMT2D* (2.4%) in breast cancer [178]. Furthermore, a range of epigenetic changes, such as restrictive heterochromatin and silencing linked to promoter DNA hypermethylation, in these two genes have been associated with cancer [179,180]. Moreover, heterozygous germline inactivating mutations in *KMT2D* have been linked to several cancers, and decreased germline activity of KMT2D was found in various pediatric cancers. In such cases, epigenetic modifications, along with other driver mutations, would be involved in cell transformation related to misregulated critical signaling pathways controlled by KMT2D [170,181,182]. Heterozygosity of a great number of mutations is common in *KMT2C* and *KMT2D* genes. As mentioned earlier, these mutations involve frameshifts, indels (insertions/deletions), and truncation of the SET domain and lead to the inactivation of these proteins. It was concluded that a wt allele of the *KMT2C* and *KMT2D* genes is crucial to sustain the viability of cancerous cells, as a total lack of their homozygous inactivating mutations was observed in the malignancies with sequenced genomes [171].

*KMT2C* is a well-established haploinsufficient tumor-suppressor gene localized on 7q and is frequently deleted in a hemizygous manner in AML patients [165,183]. It was observed that a ~50% reduction in the gene dosage by KMT2C shRNA in hematopoietic stem and progenitor cells (HSPCs) transplanted to mice triggered the development of leukemia. A similar effect—initiation of leukemia—was demonstrated through the CRISPR/Cas9 suppression of *KMT2C* in HSPCs in a heterozygous context, where one *KMT2C* allele was wt and the other was mutated. However, it needs to be noted that the suppression of *KMT2C* alone does not suffice to induce leukemogenesis, and the cellular background also plays a role, including p53 loss (p53^−/−^ HSCPs), which cooperates with *KMT2C* to impair the differentiation of these cells, leading to a myelodysplastic syndrome (MDS)-like state. Another important feature of 7q deletions in patients with MDS and AML is the loss of other genes that could contribute to tumor suppression. The question of whether these actions of *KMT2C* and other genes are additive, synergistic, or redundant needs to be answered in future research [165]. A study demonstrated that a loss-of-function mutation in the SET domain within the *KMT2C* allele in *MLL3*^Δ/Δ^ mice led to the development of ureter epithelial tumors [36]. Furthermore, the restoration of *KMT2C* expression reduced the growth of colorectal cancer (CRC) cells and reinforced the genome-wide H3K4me1 mark at the enhancers, which also suggests that inactivated KMT2C could promote CRC development through transcriptional dysregulation [184].

As already mentioned, KMT2C and KMT2D exert their activities at gene enhancers and are generally considered as haploinsufficient tumor suppressors in normal cells. Mutations in *KMT2D* lead to the misregulation of KMT2D-dependent enhancers, and their effects might be context- and cell type-dependent. Thus, for KMT2D, a more complex cell-specific situation has to be described. KMT2D was found to be involved in many important cellular pathways, such as the p53 signaling pathway and cAMP-mediated signaling, and the promotion of retinoic acid-responsive gene transcription, as demonstrated by the global identification of KMT2D-targeted loci [185]. Moreover, KMT2D interacts directly with p53, thereby promoting the expression of its target genes, which indicates the role of KMT2D in the p53 tumor-suppression pathway. As demonstrated by cell-based assays, the COMPASS-like complex via NCOA6, a multifunctional coactivator, and KMT2C (or its paralog KMT2D) act as p53 coactivators and are required for H3K4 trimethylation and the expression of p53 target genes in response to doxorubicin, a DNA-damaging agent. It was shown that the downregulation of both KMT2C and KMT2D is needed to suppress the doxorubicin-inducible expression of several p53 target genes. These results revealed the redundancy between these two KMT2s [36]. The antitumor activity of KMT2D was also analyzed in a study in which the brain-specific knockout of *KMT2D* in mice resulted in spontaneous medulloblastoma. The study showed that KMT2D was responsible for establishing super-enhancers and broad H3K4me3 peaks at tumor-suppressor genes, such as *Dnmt3a* and *Bcl6*, and activating their expression. The actions of DNMT3 and Bcl6 led to the inhibition of the oncogenic Ras and Notch pathways, respectively [186]. KMT2D is also identified as a lung tumor suppressor. It is frequently mutated in lung cancer, and its lung-specific loss in mice was demonstrated to promote lung cancer by impairing the super-enhancers, including the super-enhancer of the circadian rhythm repressor PER2, which regulates many glycolytic genes [187].

On the other hand, a mutated p53 also directly interacts with KMT2D, promoting its recruitment to gene enhancers required for TNFα-inducible H3K4me1 and K3K27ac, as observed in colon cancer cells. This interaction was shown to regulate the aberrant activity of the enhancers and, consequently, to activate the expression of tumor-promoting genes in response to chronic TNFα signaling [188]. KMT2D also mediates the ER-dependent transcription regulated by the PI3K pathway in breast cancer [189]. Furthermore, a higher *KMT2D* expression was associated with a poor prognosis in this cancer type [190]. The downregulation of overexpressed *KMT2D* in gastric cancer cells resulted in the suppression of the proliferation and induction of apoptosis [191]. In the case of MLL-AF9-induced AML in mice, *KMT2D* was found to support this MLL-r leukemia, whereas its deletion led to enhanced myelopoiesis and myeloid differentiation and, thus, protected the animals from AML-related death [168].

In recent years, KMT2C and KMT2D methyltransferases have been shown to play an important role in genome stability and DNA repair. These, together with PTIP, a subunit of the KMT2C/KMT2D complexes, were found to increase the instability and induce the degradation of the MRE11-dependent replication fork in BRCA (breast cancer type susceptibility protein)-deficient cells. Following replication stress, the recruitment of the MRE11 nuclease to stalled replication forks was found to be dependent on KMT2C/KMT2D and PTIP and correlated with KMT2C/KMT2D-induced H3K4 methylation at replication forks in BRCA-deficient cells. It has been suggested that the loss of PTIP may confer drug resistance to these cells by restricting the access of MRE11 to stalled replication forks [192]. In addition, KMT2D was found to interact with the helicase RECQL5, which is proposed to counteract the collisions between transcription and replication machinery [193,194]. Mutations in *KMT2D* were shown to cause transcription stress and genome instability [193]. Alterations in KMT2C and KMT2D could also facilitate DNA breaks and chromosomal translocations, possibly in early replicating fragile sites located at early replicating and actively transcribed gene clusters. These regions are particularly prone to replication–transcription collisions and are the source of rearrangements occurring in cancer cells [194]. Furthermore, deficiency of the KMT2C protein in bladder cancer cells was found to be associated with the modified expression of genes involved in the DNA-damage response (DDR) and DNA repair. In particular, these cells have a smaller capacity to repair dsDNA breaks by homologous recombination. Therefore, they are more prone to genomic instability and depend on PARP1/2 for DNA repair, which could be an interesting target for treatments with PARP1/2 inhibitors such as Olaparib, discussed in a later part of this review [195].

### 5.3. KMT2F (SET1A) and KMT2G (SET1B)

Mammalian KMT2F and KMT2G catalyze H3K4me3 across the entire genome and are responsible for *bulk* H3K4me3 genome-wide [27,51,196]. Similar to other H3K4 methyltransferases, *KMT2F* and *KMT2G* are mutated in different types of human cancers, whereas missense, nonsense, and frameshift alterations most commonly occur within their SET domain. A special feature of these two KMT2s is their mutations in the N-terminal RRM in certain cancers. However, the frequency of alterations in the RRM is low—3.5% for *KMT2F* and 9.4% for *KMT2G* [146,197]. The KMT2F complex has been shown to function together with β-catenin in the regulation of Wnt target genes and control the growth of colorectal cancer (CRC) cells in vitro and in vivo [198]. Furthermore, *KMT2F* was demonstrated to be upregulated in CRC cells, as was the H3K4me3 global mark in the tissues obtained from CRC patients. The downregulation of this gene turned out to be beneficial, as it inhibited the growth of CRC cells and colony formation [198]. Moreover, *KMT2F* is commonly duplicated and overexpressed in breast cancer cells [197,199,200], and its ablation leads to the decreased in vitro migration and invasion of these cells. KMT2F induces metastasis by H3K4 methylation at proximal promoters of several different metalloproteases, which are upregulated in breast cancer cells [200]. Furthermore, KMT2F acts as a positive regulator of cell cycle progression through the miRNA network. It has been found to suppress the expression of *BTG2*, a p53-inducible antiproliferative gene, by inducing several BTG2-targeting microRNAs (miRNAs) [201]. In other studies, this H3K4 methyltransferase was demonstrated to promote the growth of liver cancer cells [202] and induce proliferation by regulating several mitosis-related and DDR genes [203]. Interestingly, KMT2F also exerts its oncogenic effect by methylating substrates other than histones. For instance, it was shown to methylate YAP, an effector of the Hippo tumor-suppressor pathway and, thus, regulates the activity of YAP and promotes tumorigenesis [88]. KMT2F has also been demonstrated to methylate HSP70. The KMT2F-catalyzed dimethylation of HSP70 at Lys-561 is specifically increased in cancer cells and regulates the subcellular localization of HPSP70. Methylated HSP70 is predominantly localized in the nucleus, where it interacts with the Aurora B kinase and enhances its activity, thus promoting the proliferation of cancer cells [89]. Taken together, KMT2F plays an oncogenic role, as it is synthesized at higher levels in many cancers compared to normal cells and sustains tumorigenesis.

*KMT2G* was shown to be upregulated in renal clear carcinoma in advanced tumor stages and metastatic cells [204]. Furthermore, an A1054del *KMT2G* mutant was found to be more potent in inducing proliferation, migration, and invasion, compared to its wt allele in primary hepatic neuroendocrine tumors, by regulating genes such as *TP53* [205]. A recent study revealed that a cytoplasmic variant of KMT2G/COMPASS plays an important role in cancer cell survival and the pathogenesis of triple-negative breast cancer (TNBC). KMT2G has been demonstrated to be overexpressed in breast cancer cells and found to be crucial in determining their viability. When KMT2G or its cytoplasmic-interacting protein BOD1 (biorientation of chromosomes in cell division protein 1) were lost from the complex, many genes modulating fatty acid metabolism, such as *ADIPOR1* (adiponectin receptor 1), *COX7C*, *SDC4*, and *COQ7*, were upregulated. These data indicate that ADIPOR1 signaling, inactivated not only in cancer but, also, in obesity, is an important target of the KMT2G complex, suggesting the novel role of SET1B/COMPASS independent of its catalytic activity. Targeting this pathway is described as one of the anticancer strategies in a later part of this review [206].

## 6. Roles of the Core Subunits in Oncogenesis

A growing body of experimental data indicates that not only KMT2 enzymes but, also, the core subunits of the KMT2 complexes are involved in oncogenesis. While KMT2 mutations are present in a diverse set of human cancers, mutations of the core subunits are rarely identified. In contrast, changes in the expression of core subunits have been found to be associated with cancer.

It should be noted that the subunits of the WRAD core complex can interact with many other proteins and are found to be a part of other complexes than KMT2. For example, the WDR5 protein binds to the NSL (nonspecific lethal) complex and may be involved in the acetylation of histone H4 [207], as well as associated with ATAC (Ada2-containing histone acetyltransferase) complexes that acetylate histone H4 at lysine 16 (H4K16) [208]. DPY30 was recently suggested to be a subunit of the NURF complex and associated with it by interacting with the BAP18 subunit [209]. These suggest that the role of the core subunits in cancer may also be related to their other activities beyond KMT2 complexes.

### 6.1. WDR5

In addition to its activating function in KMT2 complexes, WDR5 is involved in their recruitment to chromatin. It interacts with many transcription factors, histone modifications, and chromatin remodeling proteins to recruit KMT2 complexes to genomic loci. Furthermore, WDR5 has been shown to interact with many lncRNAs, such as HOTTIP and NeST, to regulate the lncRNA-mediated trimethylation of H3K4, thus controlling gene transcription. More evidence points to the tumor-promoting activity of WDR5. For instance, *WDR5* has been found to be overexpressed in a number of cancers, including AML, neuroblastoma, prostate cancer, CRC, and bladder cancer [210,211,212,213,214].

WDR5 was identified to be an important factor in the pathogenesis of AML [211]. It has been found to interact with C/EBPα-p30 (CCAAT enhancer-binding protein-α), a short, mutated form of the transcription factor C/EBPα, the expression of which is characteristic of up to 9% of AML cases. Through this interaction, WDR5 directs KMT2A to specific genomic loci regulated by C/EBPα-p30, leading to their aberrant regulation and, thus, contributing to the enhanced self-renewal and inhibition of myeloid cell differentiation.

WDR5 also interacts directly with the c-MYC and N-MYC oncoproteins and is an essential cofactor of MYC-driven oncogenesis. The MYC transcription factor binds to DNA in the MYC/MAX dimer form, which recognizes consensus sequences called E-boxes. Recent studies, however, indicate that the association of MYC with chromatin also depends on its interaction with WDR5. Mutations in the MYC protein that disrupt its interaction with WDR5 reduce MYC binding at about 80% of its target genes and its ability to promote the neoplastic process and formation of iPS cells [61,215]. Amplification of the *N-MYC* oncogene is often observed in cancers of neural origin, such as neuroblastomas, and correlates with aggressive tumor behaviors, as well as poor survival. WDR5 expression is promoted by the N-MYC oncoprotein and is increased in neuroblastoma cells, which is associated with a poor prognosis of patients. The cooperation between WDR5 and MYC in neuroblastoma involves a direct interaction between them in the promoters of N-MYC target genes such as *MDM2* and other protumorigenic genes, leading to H3K4 trimethylation and their activation [213]. RNAi-*mediated* gene silencing of WDR5 or the inhibition of the N-MYC/WDR5 complex formation by a small molecule antagonist of WDR5 inhibits the expression of N-MYC target genes, which suppresses the growth of neuroblastoma cells [213].

Increased levels of the WDR5 protein found in bladder cancer tissues also correlate with advanced tumor stage and poor survival. WDR5 has been shown to promote the proliferation of bladder cancer cells by activating the transcription of cyclin genes and the *UHMK1* gene in a manner dependent on histone H3 lysine 4 trimethylation. Moreover, the elevated levels of this protein were shown to enhance chemoresistance by upregulating the antiapoptotic *MCL1* and *BIRC3* genes in bladder cancer cells via H3K4me3 [210]. An elevated WDR5 level is also characteristic of CRC cells and is an essential factor promoting CRC metastasis. It has been shown to trigger EMT in response to the PI3K/AKT signaling pathway by directly activating the expression of the *ZNF407* gene [214].

It was also found that WDR5 binds to the polycomb Cbx8 protein, in the noncanonical PRC1 complexes, and regulates H3K4me3 on the genes of the Notch network, thus promoting breast cancer [216].

WDR5-MYC interaction also protects the cancer cells from replicative stress and DNA damage, as demonstrated in PDAC [217]. The effect of WDR5 on DDR and cancer sustaining was found in colon cancer studies, which showed that WDR5 depletion sensitized the colon cancer cells to radiation-induced DNA damage [218]. WDR5 also regulates the expression of genes involved in DNA repair, and silencing of its expression leads to an increased response to DNA damage in cells undergoing reprogramming into iPS cells [219]. Furthermore, WDR5/KMT2 complexes have been shown to confer cancer cells with resistance to genotoxic stress by promoting the expression of many genes in the glutathione (GSH) metabolic cascade [220].

The interaction of WDR5 with lncRNAs facilitates the recruitment of WDR5/KMT2A complexes and lncRNA-mediated gene transcription and determines the role of WDR5 as a tumorigenic agent in many cancers. WDR5 has been found to bind many lncRNAs, including HOTTIP, a lncRNA regulating the expression of *HOX* genes, the upregulation of which is characteristic of various types of human cancers. This interaction has been shown to regulate the properties of pancreatic cancer stem cells by promoting the expression of the *HOXA9* locus [221]. In addition, WDR5 physically interacts with the BLACAT2 (bladder cancer-associated transcript 2) lncRNA that is markedly upregulated in bladder cancer with lymph node metastasis. The association of WDR5 with BLACAT2 promotes H3K4 methylation and the upregulation of VEGF-C, a lymphangiogenic growth factor, thereby leading to bladder cancer-associated lymphangiogenesis and lymphatic metastasis [222]. Another lncRNA interacting with WDR5, GCAWKR, promotes the development of gastric cancer by upregulating the expression of the target *PTP4A1* gene [223].

The WDR5 protein may also recruit KMT2 complexes to chromatin by recognizing various histone modifications, and this mechanism may contribute to its role in cancer. WDR5 has been shown to be overexpressed in prostate cancer and involved in the proliferation of androgen-dependent prostate cancer cells. It was found to bind to H3T11P (PKN1-mediated histone H3 threonine 11 phosphorylation) upon androgen stimulation in androgen-dependent prostate cancer cells to recruit KMT2A complexes and induce the expression of androgen receptor target genes [212]. WDR5 also binds to ACK1-mediated H4Y88ph and directs KMT2D to the promoter of the androgen receptor gene, thereby promoting its expression in castration-resistant prostate cancer [224]. Recent data have demonstrated the recruitment of the WDR5/KMT2A complex to H3R2me1/H3R2me2 marks enriched by β-catenin signaling in the promoters of the GSH metabolic cascade genes in response to genotoxic stress. This contributes to the restoration of redox homeostasis and confers resistance to genotoxic stress in ovarian cancer cells.

### 6.2. RBBP5

Similar to WDR5, the expression of RBBP5 is also increased in many cancers. A study showed that RBBP5 expression is upregulated in hepatocellular carcinoma and correlates with a poor prognosis of patients [225]. Another study detected an increased level of RBBP5 in glioblastoma, which was significantly associated with the pathology grades [226]. The *RBBP5* gene silencing, performed in both studies, inhibited cell proliferation and induced the apoptosis of tumor cells.

A recent study also demonstrated that RBBP5 is essential for the maintenance of glioblastoma cancer stem cells in an inhospitable microenvironment [227]. Glioblastoma cancer stem cells exhibit a lower expression of the innate immune receptor TLR4 (Toll-like receptor 4), which helps them to evade inhibitory innate immune signaling. Since TLR4 inhibits the expression of RBBP5 by activating TBK1 kinase signaling, a lower level of TLR4 in glioblastoma cancer stem cells favors an increased RBBP5 expression, which promotes the expression of pluripotency genes [227].

### 6.3. ASH2L

ASH2L has been shown to be overexpressed at the protein level in most human tumors and tumor cell lines, and its knockdown inhibited the proliferation of tumor cell lines [228]. An analysis of its oncogenic activities using the rat embryo fibroblast (REF) co-transformation assay showed that ASH2L cooperates efficiently with activated HRAS (Harvey Rat Sarcoma Viral Oncogene Homolog) in REF transformation, which strongly suggests that ASH2L functions as an oncoprotein [228]. Consistent with these results, low levels of ASH2L expression correlated with an increased overall survival in AML patients [229]. Recent studies have also shown that ASH2L may act as a coactivator of ERα and promote the progression of endometrial cancer. ASH2L was found to interact with ERα to regulate a subset of estrogen-induced target genes, such as the *PAX2* (paired box 2) transcription factor gene, thereby promoting the proliferation and migration of endometrial cancer cells [230].

### 6.4. DPY30

DPY30, like other core subunits of the KMT2 complexes, has been found to be overexpressed in many cancers. Its increased level was detected in cervical squamous cell carcinoma [231], as well as in Burkitt’s lymphoma [232], gastric cancer [233], and ovarian cancer, and correlated with a poor prognosis of patients [234].

Studies on Burkitt’s lymphoma have shown that DPY30, like other KMT2 core subunits, is upregulated by the MYC oncoprotein and is significant for the binding of MYC to its target genes; thus, it plays an important role in MYC-driven tumorigenesis. It has been proposed that DPY30, to some extent, regulates MYC recruitment through its role in KMT2 complexes, as well as by interacting with other factors such as BPTF, a subunit of the NURF chromatin remodeling complex [232].

## 7. Therapeutic Strategies Targeting the Aberrant Activity of KMT2 Complexes in Cancers

In recent years, there has been tremendous progress in research aiming to find molecules that can block the activity of H3K4 KMT2 complexes or their downstream pathways, thus abrogating the oncogenic phenotype. Efforts are made to find chemicals that can be used to treat leukemias caused by KMT2A rearrangements. These inhibitors can act on proteins recruited to the MLL-FP complexes or wt KMT2A needed to maintain the leukemic state of cells transformed by the actions of MLL-FPs. The histone methyltransferase activity of KMT2A can be blocked by chemicals capable of disrupting KMT2A interactions with the core proteins such as WDR5 [235]. Targeting wt KMT2A activity can be used not only in MLL but, also, in MDS and AML, in which KMT2A amplification or tandem duplication is detected. Among the interactions within the MLL-FP complexes, the main targets are DOT1L and SEC [236,237,238]. Other subunits, such as Menin or LEDGF, which function in both the MLL and MLL-FP complexes, can also be targeted [236,239]. Furthermore, many other strategies were described, such as the inhibition of KMT2A protein degradation [240] and metabolic pathways [187] or a modified chimeric antigen receptor (CAR) T-cell therapy [241]. Molecular blockers can inhibit the subunits of MLL-FP complexes such as KMT2A-ENL to downregulate *MYC* expression, which is crucial for MLL-r leukemia. Another approach is the inhibitory targeting of proteins such as BRD4 (bromodomain-containing protein 4) recruited to the regulatory elements of the *MYC* gene, which can switch off the MYC-dependent leukemia sustainment program [236,242,243,244].

One inhibitor that has been developed to block the activity of KMT2A is MM-401, a macrocyclic peptidomimetic that antagonizes the interaction of WDR5 and wt KMT2A and, thus, inhibits H3K4 methylation and promotes myeloid differentiation in MLL cells. The action of MM-401 phenocopies *KMT2A* gene deletion and has no toxicity on normal bone marrow cells or an inhibiting effect toward non-MLL cells [245]. Another interesting example is the chemical compound OICR-9429, which disrupts the interaction between WDR5 with MLL and was demonstrated to reduce the recruitment of KMT2A at the loci occupied by a p30 (also known as C/EBPα) translational isoform expressed in AML. Through selective inhibition of the proliferation and induction of differentiation, OICR-9429 was found to kill patient-derived AML cells expressing p30 [211]. Another approach using this WDR5 inhibitor involved the p53 pathway. GOF alteration in the *TP53* gene led to an increased expression of KMT2A and KMT2D, which, in turn, enhanced genome-wide H3K4 mono- and trimethylation and cancer cell proliferation. Targeting KMT2A complexes with OICR-9429 was shown to inhibit the growth of cancer cells containing *TP53* GOF mutations [154]. The list of WDR5 inhibitors disrupting its interaction with KMT2A and N-MYC or compounds inhibiting the arginine-binding cavity of WDR5, also known as the WIN (WDR5-INteraction) site, and displaying antioncogenic activities has been growing in recent years [211,213,217,246,247,248].

Interesting approaches to block protein–protein interactions within COMPASS-like complexes for inhibiting cancer growth were also proposed for Menin-KMT2A, and several disruptors such as MI-463, MI-503, and M-525 were described [249,250]. Menin interacts directly with LEDGF via its integrase-binding domain [21,251]. LEDGF, as a part of the MLL-FP complexes, switches a physiological H3K4me3 to an aberrant cancer-related H3K36me2 [252,253]. It has become a potential therapeutic target due to its essential role in MLL-r leukemia and nonessential involvement in hematopoiesis [254,255]. However, up until now, there has been no real success in implementing LEDGF inhibitors, including a cyclic peptide CP65, for the treatment of MLL-r leukemia [256].

DOT1L is recruited to the MLL-FP complexes with fusion proteins such as ENL, AF9, and AF10 [257]. As already explained, DOT1L is responsible for H3K79 methylation in leukemia carried out by MLL-FP complexes [258]. Therefore, research was performed to obtain small molecules with selective inhibitory properties targeting DOT1L, which resulted in the identification of a chemical compound, named EPZ004777, capable of inhibiting DOT1L-dependent H3K79 methylation and downregulating the expression of leukemogenic genes [237]. A similar activity is displayed by another chemical agent, EPZ-5676 (pinometostat) [259]. However, its modest potential as an anticancer drug was not promising, as concluded by a clinical investigation in patients with adult acute leukemia [238]. It is of interest that DOT1L can also be targeted in the leukemias devoid of KMT2A rearrangements [260]—or, even, in the KMT2-unrelated solid tumors—to suppress the proliferation and metastasis of breast cancer cells [261].

One crucial transcription factor, the expression of which is regulated by MLL-FP complexes and is essential for sustaining leukemogenesis, is MYC. However, it has a three-dimensional structure that is difficult to be reached by drugs. Therefore, the activity of MLL-FP complex subunits—namely, BRD4, CBP/p300, and ENL, which interact with the *MYC* regulatory elements—is targeted. A chemical compound named I-BET151 displaces BRD4 from chromatin, thereby downregulating *BCL2*, *CDK6*, and *MYC*, and shows a potent antileukemic activity in MLL-r cells and in vivo [236,242,262]. Another approach involved the use of A-485, an inhibitor of the catalytic core of CBP/p300, which turned out to have a very potent anticancer activity against many tumor cells, including MOLM-13 MLL-r leukemic cells [263]. ENL contains a YEATS domain, which is a reader of lysine acetylation (Kac) [264] and was demonstrated to be crucial for the maintenance of MLL-r leukemias. The depletion of ENL by Cas9 in leukemic mice xenotransplanted with MV4-11 or MOLM-13 cells led to the downregulation of key leukemic drivers such as MYC and the prolonged survival of mice [265,266]. Later, several compounds were found to inhibit the YEATS domain of AF9 and ENL and downregulate many leukemic driver genes [267,268].

In general, monotherapies are rarely efficient, and the administration of a single agent does not provide the expected therapeutic effect in cancer treatments. Therefore, many researchers have demonstrated that a combination of compounds can improve the antioncogenic outcome. Such synergy was shown in human leukemic cells with SGC0946 and I-BET molecules inhibiting DOT1L and BRD4, respectively [269]. The above-mentioned EPZ004777 and MI-2-2, a second-generation inhibitor of KMT2A-Menin interactions [270,271], are more potent together in downregulating crucial leukemic genes such as *MYC*, *HOXA9*, and *MEIS1* and reducing the proliferation of MLL-r leukemic cells [272]. Another strategy is the downregulation of *MYC* by CBP/p300 inhibition at its bromodomain using I-CBP112, which inhibits the growth of cancer cells in human AML cell lines with *MLL-AF9* translocation. The compound was also able to sensitize MOM-13 MLL-r leukemic cells to JQ1, mentioned earlier, and the combination of these two agents resulted in a better antileukemic outcome [273].

Recent studies have shown that stabilizing the wt KMT2A protein in leukemic cells could have therapeutic potential. This would displace the more stable and abundant KMT2A-FPs from chromatin, which, in turn, would abrogate the oncogenic phenotype of these cells. Such an action could be performed by targeting the pathway of UBE2O and interleukin 1 (IL-1) responsible for the degradation of the KMT2A protein. It has been demonstrated that the inhibition of IL-1 receptor-associated kinases (IRAK) has a negative impact on the proliferation of MLL-r leukemic cells both in vitro and in vivo. This new approach of targeting MLL-FP complex degradation pathways could be used for the treatment of MLL-r leukemia, which is aggressive and resistant to other types of therapeutic strategies. It could also be potentially applied in the treatment of other cancer types derived from gene translocation by stabilization of the wt allele [240].

Another interesting approach to treat leukemias and lymphomas is chimeric antigen receptor (CAR) T-cell therapy. In MLL-AF4 B-cell acute lymphoblastic leukemia (ALL or B-ALL), the expression of the *PROM1* gene encoding a specific marker (CD133) is regulated by MLL-AF4 and has proven to be a good target for the treatment of MLL-r leukemia [274,275]. The rationale to target CD133 was an acquisition of a CD19-negative myeloid phenotype, allowing for the immune escape of MLL-r B-ALL from CD19 CAR T-cell therapy in some patients [276,277]. A recent promising study showed a novel bispecific CD19/CD133 CAR strategy, which can target both CD19 and CD133 [278]. In vitro assays demonstrated its robust cytotoxicity against CD19^+^CD133^+^ and CD19^−^CD133+ B cells, which could help reduce the subsequent lineage switch in MLL-r B-ALL [278]. However, the following concerns were raised. The expression level of *PROM1* is similar in both MLL-r B-ALL primary blasts and normal nonlymphoid HSPCs. Therefore, “on-target, off-tumor” toxic effects can be expected, as CD133 can be targeted all the time independently of the CD19 co-expression. Several interesting solutions to overcome these issues were proposed [279]. Thus, CAR T-cell therapy is certainly promising if its limitations are overcome in the upcoming years [241].

A great number of molecular strategies were designed for the treatment of KMT2A-related leukemias; however, progress has also been made in other KMT-related cancers in recent years. It is quite impossible to target the activity of tumor suppressors such as KMT2C/D, which are most commonly truncated in various cancers [170]. Instead, histone-modifying enzymes involved in transcriptional repression can be blocked, which helps restore the normal gene expression pattern in *KMT2C* mutant cells [174]. One such enzyme is EZH2, responsible for H3K27me3, which is a subunit of the polycomb repressive complex 2 (PRC2) [280], which can be blocked by using a GSK126 agent. KMT2C was also demonstrated to participate in DDR, and bladder cancer cells having low KMT2C activity cannot efficiently repair the dsDNA breaks by homologous recombination. Therefore, PARP1/2 inhibitors were proposed as antioncogenic agents for KMT2C-associated cancers [195]. A recent study demonstrated that a *KMT2D* loss decreased the synthesis of PER2 (Period Circadian Regulator 2), which is involved in the regulation of many glycolytic genes. Thus, pharmacologically contained glycolysis helped repress the tumorigenicity of human lung cancer cells with an inactivated *KMT2D* gene [187].

While designing inhibitory strategies, much focus was directed to the other roles of H3K4 methyltransferases [281]. For example, KMT2G, regardless of its SET domain, is crucial for suppressing ADIPOR1 cytoplasmic signaling, which induces an oncogenic outcome [206]. As this signaling is very important in TNBC, a previously identified activator of AMPK and PPAR-α (peroxisome proliferator-activated receptor alpha) pathways and agonist of ADIPOR1, named AdipoRon [282], was proposed as a potential agent for the treatment of TNBC [206], as well as other cancers such as pancreatic malignancies, which are KMT2G-unrelated [283].

Although the core subunits of KMT2 complexes such as WDR5, RBBP5, DPY30, and ASH2L are rarely deleted or carry genetic mutations, they are frequently amplified or upregulated in human cancers and act as oncogenes [218,228,230,232,284]. Therefore, they could constitute potential targets in molecular therapies for cancer patients. Since WDR5 is only required for the catalytic activity of KMT2A complexes and RBBP5 is an obligatory component regulating all other KMT2 complexes, it could be assumed that RBBP5 antagonists may exert broader inhibitory actions compared to agents targeting WDR5 [146,245,285,286]. DPY30 was found to be upregulated in Burkitt’s lymphoma, and its downregulation can have a negative effect on cell transformation dependent on MYC activity. Therefore, DPY30 and H3K4 methylation were suggested as potential epigenetic pathways that could be therapeutically targeted in MYC-dependent cancers [232,284]. DPY30 can be targeted using cell-penetrating peptides (CPPs) derived from ASH2L, which normally binds DPY30 in MLL-FP complexes. The disruption of DPY30-ASH2L interaction by CPPs turned out to be effective in inhibiting the growth of MLL-r leukemic cells and other hematologic cancers that were dependent on MYC activity [287]. The specificity and actions of the chosen inhibitors upon MLL and MLL-FP complexes are depicted in Table 2.

## 8. Conclusions and Perspectives

Recent studies have helped us better understand the structure of the COMPASS complexes and the range of their methylating activities. In addition, the important roles of H3K4 methyltransferases and the core subunits in H3K4me1/2/3 regulation were deciphered to a significant extent. KMT2s are frequently mutated in hematological, as well as a broad range of nonhematological, cancers. In MLL-r leukemia, these alterations are mostly translocations of KMT2A to a number of fusion genes, while, in other cancers, they represent heterozygous nonsense mutations generating a truncated version of KMT2s. Genetic studies performed in mice proved that a loss of the wt allele of KMT2s does not lead to tumorigenesis and that haploinsufficiency alone does not drive cell transformation, and other oncogenic events are also necessary. Cancer genome sequencing has provided us with enormous data; however, it does not answer whether the observed mutations result either in gain- or loss-of-function phenotypes and whether these mutations are recessive or dominant or drivers or passengers. Therefore, additional functional studies are needed to distinguish these cellular outcomes. Mouse models and genome-editing approaches in the study of KMT2-dependent carcinogenesis are expected to greatly improve our knowledge in this field. Another important issue is the function of the *KMT2A* wt allele in MLL-r leukemias. This also applies to other cancers harboring at least one copy of a given *KMT2A* wt allele while the other is mutated. Understanding the interplay between the translocated/mutated and the wt allele will be crucial for the further development of therapeutic strategies.

Essential components of the KMT2 complexes are the core subunits, which tightly regulate the activity of the COMPASSes. One of them, the WDR5 protein, has drawn a lot of attention, and many different chemicals have been designed to disrupt its function and suppress the leukemogenic phenotype. Moreover, several new targeting strategies have emerged in recent years for the treatment of KMT2-related cancers, and although they still need to be improved, some of them have already been adopted in clinical applications; for example, CAR T-cell therapy is used for the treatment of MLL patients. It can be expected that our expanding knowledge of all KMT2 functions in oncogenesis will provide new, interesting insights that can be helpful in designing even better targeting strategies with great therapeutic potential for cancer patients.

## Figures and Tables

**Table 1:** Subunit composition of the mammalian KMT2 complexes.

	KMT2A or KMT2B Complex	KMT2C or KMT2D Complex	KMT2F or KMT2G Complex
Enzyme	KMT2A or KMT2B	KMT2C or KMT2D	KMT2F or KMT2G
Core subunits	ASH2L RBBP5 WDR5 DPY30	ASH2L RBBP5 WDR5 DPY30	ASH2L RBBP5 WDR5 DPY30
Unique subunits	Menin HCF1 or HCF2	PTIP PA1 NCOA6 UTX	CFP1 WDR82 HCF1

KMT, histone–lysine N-methyltransferase; ASH2L, absent, small, or homeotic 2-like; RBBP5, retinoblastoma-binding protein 5; WDR5, WD repeat-containing protein 5; DPY30, Dumpy-30; HCF1, host cell factor 1; PTIP, PAX transactivation-domain interacting protein; PA1, PTIP-associated 1; NCOA6, nuclear receptor coactivator 6; UTX, ubiquitously transcribed tetratricopeptide repeat, X chromosome; CFP1, CXXC finger protein 1; and WDR82, WD repeat-containing protein 82.

**Table 2:** Selected inhibitors targeting the aberrant activity of KMT2 complexes in cancer. Several major targets can be distinguished, including the core subunits (DPY30, RBBP5, ASH2L, and WDR5); LEDGF and Menin present in both wild-type (wt) MLL and MLL-fusion protein (FP) complexes; and “additional” proteins such as DOT1L, BRD4, CBP/p300, and fusion proteins (ENL) present in MLL-FP complexes. Other approaches to inhibit the actions of the mutated KMT2s, such as KMT2C and KMT2D, in cancer involve the use of repression complex blockers or contained glycolysis. More details are provided in the main text. TNBC: triple-negative breast cancer; IRAK: IL-1 receptor-associated kinase; YEATS domain: Yaf9, ENL, AF9, Taf14, Sas5 domain; PARP1/2: Poly(ADP-ribose) polymerase 1/2.

Mode of Action	Name of Inhibitor	Cellular Outcome	Targeted Cancer Cells	References
Targeting core subunits of COMPASS:	MM-401 (microcyclic peptidomimetic)	myeloid differentiation/phenocopying KMT2A deletion	MLL-r leukemia cells in culture	[245,247]
Antagonizing the interaction of WDR5 and KMT2A	OICR-9429 (small-molecule antagonist)	Inhibition of proliferation and induction of differentiation	Patient-derived AML cells expressing p30	[211]
		Inhibition of cancer cell growth	Various tumor cells with a *TP53* gain-of function (GOF) mutation	[154]
Targeting DPY30	Cell penetrating peptides (CPPs) derived from ASH2L	Inhibition of cancer cell growth	MLL-r leukemia cells/other MYC-dependent hematologic cancers	[287]
Blocking interaction of Menin with KMT2A	MI-463, MI-503 (small-molecule antagonist)	Inhibition of progression of MLL leukemia in vivo vs. normal hematopoiesis	MLL-r leukemia cells/mouse model of MLL leukemia	[249]
	M-525 (small-molecule antagonist)	Suppression of MLL-regulated gene expression, leukemia cell growth inhibition	Various cell lines derived from MLL-r leukemia (MV4, MOLM-13, MOLM-14)	[250]
	MI-2-2 (small-molecule inhibitor)	Inhibition of cell proliferation, downregulation of differentiation	MLL leukemia cells (KMT2A-F4 translocation)	[270,271]
Blocking interaction of KMT2A with LEDGF	CP65 (cyclic peptide)	Impairment of clonogenic growth of primary murine MLL-AF9-expressing leukemic blasts	MLL-AF9 leukemia cells	[256]
Targeting DOT1L in KMT2A-rearranged complexes	EPZ004777 (S-adenosylmethionine-competitive inhibitor)	Downregulation of leukemic genes, inhibition of H3K79, inhibition of proliferation	Leukemia cells bearing MLL-r/extension of survival in a mouse MLL xenograft model or complete tumor regression	[237,288]
	EPZ-5676 (pinometostat)			[259]
Dissociation of interacting proteins from MYC regulatory elements	I-BET (via BRD4)	Downregulation of MYC-regulated gene expression, inhibition of proliferation	Hematological cancers (MLL-r leukemia)	[242,262,289]
	A-485 (via the catalytic core of CBP/300)	Inhibition of proliferation	Lineage specific tumor cells (hematological and prostate)	[263]
	Inhibitors of YEATS domain of AF9 and ENL (XL-13m)	Downregulation of leukemic gene drivers	MLL-r leukemia cells	[267,268]
Stabilization of wt KMT2A	IRAK1/4	Inhibition of cancer cells proliferation in vitro/in vivo	MLL-r leukemia cells	[240,290]
Restoration of normal gene expression in KMT2C mutant cells	GSK 126 (via a subunit of the polycomb repressive complex 2)	Impairment of cell proliferation, resetting the epigenetic balance of polycomb and compass function	Cells/tumors bearing mutations (PHD domain) in KMT2C	[174]
PARP1/2-depdenent DNA repair	Olaparib (PARP1/2 inhibitor)	Synthetic lethality of cancer cells	Cancer cells with low KMT2C levels (bladder cancer)	[195]
Pharmacologically contained glycolysis	Glycolytic inhibitors (2-deoxy-D-glucose (2-DG)	Impediment of tumorigenic growth	Lung cancer cells with KMT2D mutations	[187]
Suppression of ADIPOR cytoplasmic signaling	AdipoRon (ADIPOR agonist)	Induction of cancer cell death through necroptosis	MIA PaCa-2 tumor cells/cancer cells isolated from patients with pancreatic cancer/TNBC cells	[206,282,283]
